# Serum Anti-Müllerian Hormone Levels Were Negatively Associated With Body Fat Percentage in PCOS Patients

**DOI:** 10.3389/fendo.2021.659717

**Published:** 2021-06-04

**Authors:** Er Luo, Jinxiao Zhang, Jiahui Song, Di Feng, Yaxin Meng, Hongyu Jiang, Da Li, Yuanyuan Fang

**Affiliations:** Center of Reproductive Medicine, Shengjing Hospital of China Medical University, Shenyang, China

**Keywords:** body fat percentage, anti-Müllerian hormone, polycystic ovary syndrome (PCOS), obesity, serum

## Abstract

**Background:**

Obesity is a state of excess body fat accumulation, and appears to be closely associated with polycystic ovary syndrome (PCOS). Notably, plausible biological pathways through which obesity can regulate anti-Müllerian hormone (AMH) production have been proposed, and women with PCOS characteristically have an increased AMH level. Body fat accumulation can be described by body fat percentage (BFP). However, the relationship between BFP and AMH still remains unclear.

**Materials and Methods:**

A total of 87 controls and 156 PCOS patients were divided into lean and overweight/obese groups, and the PCOS patients were further divided into hyper-AMH and normal-AMH subgroups. Univariate regression was used to assess the unadjusted relationship between AMH and outcome variables, multivariable regression analysis was performed to test whether and how serum AMH levels were associated with BFP after adjusting for other co-variables. Receiver-operating characteristic (ROC) curve analyses were used to test the utility of BFP for the diagnosis of PCOS.

**Results:**

BFP was higher in PCOS patients compared with controls, regardless of obesity. Serum AMH levels were negatively associated with BFP in the PCOS group (r = -0.371; *P* < 0.001) but not in the control group (r = -0.095; *P* = 0.385). Multivariable logistic regression analysis showed that elevated BFP was associated with a high risk of PCOS (odds ratio, 1.290; 95% confidence interval, 1.084–1.534, *P* = 0.004). Furthermore, the combination of BFP and serum AMH into a multivariate model gave an improved area under the curve (AUC) of 88.5%, with a sensitivity of 72.4% and specificity of 87.4%; the positive and negative predictive values were 91.2% and 63.9%, respectively. One limitation of this study is all the conclusion reported was based on small sample size.

**Conclusions:**

Herein, we described the negative correlation between BFP and serum AMH levels for the first time, and the present results highlight the importance of further investigation into the role of BFP, especially in body fat-related AMH change as it relates to the underlying pathogenesis of PCOS.

## Introduction

Polycystic ovary syndrome (PCOS) is a common endocrine disorder that affects up to 5%–20% of women of reproductive age; and is characterized by hyperandrogenism, ovulatory dysfunction, and polycystic ovarian morphology (1). The metabolic abnormalities often associated with PCOS—obesity, insulin resistance, hyperinsulinemia, and dyslipidemia—are not included in the definition of the syndrome because whether they are intrinsic to it is still unclear (2).

Obesity, a state of excess body fat accumulation, appears to be closely associated with PCOS (3).

International definitions of obesity for adults are based on body mass index (BMI) (4), which is calculated using the following formula: total body weight (kg)/[height (m^2^)]. However, BMI is an imperfect measure of body fat, largely because it does not directly measure fat mass (5). Body fat accumulation can be described by body fat percentage (BFP), which refers to the proportion of body fat weight in the total body weight. Studies focusing on the relationship between fat and anti-Müllerian hormone (AMH) cannot be found to date. Obesity could impair AMH production through clearly biologically plausible pathways (6). Women with PCOS have characteristically increased AMH levels (7). AMH is a dimeric glycoprotein and a member of the transforming growth factor β (TGF- β) family of growth and differentiation factors (8) that is specifically expressed in granulosa cells of small growing follicles (9). Several studies have shown that AMH expression remains high until a follicle reaches a diameter of approximately 8 mm (10). It was introduced as a surrogate measure of polycystic ovaries and as a biomarker of PCOS because of its associations with other PCOS criteria, including oligomenorrhea and hyperandrogenism (11, 12). Serum AMH levels were higher in anovulatory patients with prominent PCOS than in women without PCOS even though they also exhibited elevated levels (11). Positive correlations between serum concentrations of AMH and testosterone in PCOS have been reported (13). In addition, AMH levels have been observed to be significantly lower in obese and overweight women compared with normal-weight women (12).

The BMI has the advantage of simplicity in epidemiological studies, but it has deficiencies since it does not separate fat from lean body mass (14). The relationship between BFP and AMH and the role of BFP in PCOS are largely unknown. Understanding the BFP–AMH relationship has important implications for the clinical interpretation of laboratory values because changes in the non-serum indicator may influence the serum indicator and could illuminate the underlying ovarian physiology. Thus, the present study measured BFP and serum AMH levels from PCOS patients and control subjects with the aim of investigating the interaction between the two indicators.

## Materials and Methods

### Patients and Blood Samples

Chinese Han women aged 25–40 years were recruited from Shengjing Hospital of China Medical University, comprising 87 controls without PCOS and 156 PCOS patients. The diagnosis of PCOS was made according to the Rotterdam criteria (15). The control group was made up only of women who do not have any manifestations of the Rotterdam criteria. Patients with the following criteria were excluded from this study: polycystic ovary (PCO) morphology (presence of 12 or more follicles in each ovary measuring 2–9 mm in diameter and/or increased ovarian volume with ovarian ultrasound), pregnancy, lactation, hormonal medication treatment, smoking, inflammatory diseases, hyperprolactinemia, any other metabolic disorders such as diabetes, Cushing’s syndrome, congenital adrenal hyperplasia, androgen-secreting tumor or a history of any known neoplastic disease, and weight change of 5 kg or more in the previous six months. (**Supplementary Figure 1**). Venous blood samples were obtained after overnight fasting for at least 12 hours, during the 3rd to 5th (early follicular phase) days of spontaneous menses for non-PCOS patients or progestin-withdrawal bleeding for PCOS patients. All blood samples were centrifuged for 15 minutes at 3000 r/min and serum samples were then stored at −80°C. Samples were clotted for two hours at room temperature before centrifugation for 15 minutes at 1000 r/min for assessment of the hormone levels. All serum were centrifuged after thawing before assessment of the hormone levels. No repeated freeze-thaw cycles occurred.

To systematically study the relationship between AMH and BFP, the PCOS and control groups were divided into two categories by BMI using Asian guidelines (16): lean groups (BMI < 23 kg/m^2^) and overweight/obese groups (BMI ≥ 23 kg/m^2^), because there is no international BFP guideline for lean and overweight/obese categorizations. In this study, obesity refers to both overweight and obese patients. The PCOS patients were further divided into hyper-AMH and normal-AMH groups. PCOS patients with serum AMH ≥ 9.34 ng/mL, representing the 95th percentile of basal serum AMH in the control group, belonged to the hyper-AMH group. Characteristics of the subjects are provided in **Supplementary Table 1**.

This study was approved by the Institutional Review Board at China Medical University on 28th February 2015 (reference number 2015PS108K), and informed consent was obtained from each of the patients before the study.

### Measurement of Clinical and Biochemical Indicators

BFP, waist-hip ratio, fat mass, and visceral fat were measured with tight clothes using the Body Composition Analyzer BCA-1C, which uses the bioelectrical impedance method. The resistance range was 100–1000 Ω.

Blood samples were analyzed for lipids, along with insulin and glucose levels, by semi-automated enzymatic methods, whereas total testosterone, progesterone, follicle-stimulating hormone (FSH), thyroid-stimulating hormone (TSH), luteinizing hormone (LH), estradiol, prolactin, and were assayed using a chemiluminescence analyzer. All AMH values were assayed with a chemiluminescence kit from Beckman Coulter (B13127-Access AMH reagent). The reportable measuring range was 0.02 ng/mL to 24 ng/mL, the limit of detection was ≤ 0.02 ng/mL, and the limit of quantitation was ≤ 0.08 ng/mL. Serum free testosterone (CSB-E05096h, Cusabio Biotech, Wuhan, China) and human dehydroepiandrosterone sulfate (DHEA-S; CSB-E05105h, Cusabio Biotech, Wuhan, China) were measured using commercial ELISA kits following the manufacturer’s protocol. The assay sensitivity limits for detecting free testosterone and DHEAS were described by the manufacturer as 1.2 pmol/L, 3.7 pg/mL and 10 ng/mL, respectively. The intra-assay and inter-assay coefficients of variability (CVs) were all 15%. Serum concentrations of sex hormone-binding globulin (SHBG) (Human SHBG ELISA Kit; RayBiotech, Norcross, GA, USA) were measured using commercial ELISA kits following the manufacturer’s protocol too, the intra-assay CVs were 10%, and the inter-assay CVs were 12%. Concentrations were determined by comparing the optical densities (450 nm) of samples with the standard curve.

### Statistical Analysis

Data were expressed as the mean ± standard deviation or median (interquartile range) as appropriate. The Kolmogorov-Smirnov test was used to test the normality of distribution of the continuous variables. The comparisons between groups were performed using analysis of Student’s t test for two normally distributed independent samples and one-way analysis of variance (ANOVA) for multiple groups. The Mann-Whitney U test was used for non-normally distributed variables. The non-normally distributed multiple groups were logarithmically transformed (log_10_) before statistical analysis. Correlations between BFP and AMH were tested with Pearson analysis. Univariate linear regression was used to assess the unadjusted relationships between AMH and outcome variables, and multivariable linear regression analysis was used to test whether and how serum AMH levels were associated with BFP after adjusting for other co-variables. Multivariable logistic regression was used for assessing the strength of the association between BFP and PCOS after adjusting other co-variables. Lastly, receiver operating characteristic (ROC) curves were prepared to compare the diagnostic performance of AMH and BFP parameters, individually or in combination. The area under the ROC curve (AUC) with 95% confidence interval, sensitivity, and specificity for the diagnosis of PCOS were calculated. The optimal cutoff point was calculated by Youden index. All statistical analyses were performed using SPSS for Windows version 26. All tests were two-sided, and P < 0.05 was considered statistically significant.

## Results

### Differences in BFP and AMH in PCOS and Control Groups

**Figure 1:**
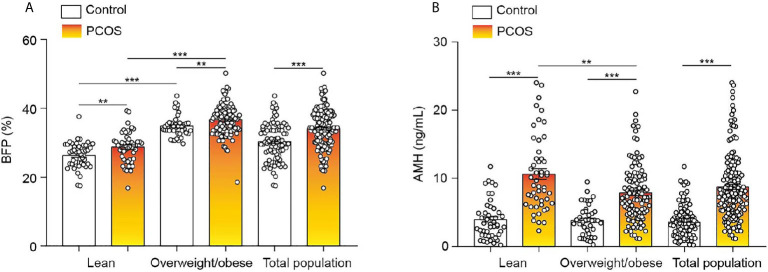
BFP and serum AMH levels of the control and PCOS groups. **(A)** Differences in BFP between lean and overweight/obese control and PCOS groups. **(B)** Differences in AMH levels between lean and overweight/obese control and PCOS groups. Bar graphs show the standard error of mean. ***P < 0.001, **P < 0.01. PCOS, polycystic ovary syndrome; BFP, body fat percentage; AMH, anti-Müllerian hormone.

In general, BFP was significantly higher in the PCOS group than in the control group (**Figure 1A**), and similar results were seen for AMH (**Figure 1B** **)**. To explore these observations further, the control and PCOS groups were divided into lean and overweight/obese groups. As presented in **Table 1**, in the lean groups, although BMI was not different between the PCOS and control groups, BFP and serum AMH levels were higher in the PCOS group. Similar outcomes were found in the overweight/obese groups. In addition, waist–hip ratio and fat mass were greater in the lean PCOS group than in the lean controls, and visceral fat was higher in the PCOS group with obesity. The lean PCOS group had higher serum AMH levels than the overweight/obese PCOS group; no significant differences were observed in the control groups (**Figure 1B**).

**Table 1:** Description of the study participants categorized by BMI.

	Lean (BMI < 23 kg/m^2^)			Overweight/Obese (BMI ≥ 23 kg/m^2^)		
	Control (n = 46)	PCOS (n = 50)	*P*-value	Control (n = 41)	PCOS (n =106)	*P*-value
Age (year)	30.67 ± 3.46	29.48 ± 2.94	0.071	31.61 ± 3.01	30.72 ± 3.10	0.117
BMI (kg/m^2^)	20.45 ± 1.58	20.94 ± 1.55	0.123	26.32 ± 2.97	27.13 ± 2.77	0.123
BFP (%)	26.30 ± 3.77	28.73 ± 4.39	0.005	34.81 ± 3.18	36.68 ± 4.17	0.004
Waist-hip ratio	0.84 (0.80–0.86)	0.85 (0.83–0.87)	0.024	0.92 (0.90–0.94)	0.93 (0.91–0.97)	0.210
Fat mass (kg)	14.18 ± 2.95	15.64 ± 3.97	0.046	24.77 ± 5.91	26.22 ± 6.58	0.221
Visceral fat (kg)	8.05 (6.78–8.90)	8.30 (7.08–9.13)	0.083	11.20 (10.60–12.05)	12.05 (10.88–14.00)	0.029
AMH (ng/mL)	3.91 ± 2.82	10.62 ± 5.53	0.001	3.81 ± 2.21	7.85 ± 4.27	0.001
Total T (ng/mL)	0.49 ± 0.19	0.70 ± 0.29	0.001	0.43 ± 0.19	0.63 ± 0.22	0.001
Free T (nM)	0.02 (0.02–0.03)	0.03 (0.02–0.04)	0.002	0.02 (0.02–0.03)	0.03 (0.02–0.05)	0.001
DHEAS (nM)	2485.70 (1872.11–3586.00)	3778.64 (2655.18–5321.61)	0.826	2345.58 (1791.56–3278.80)	4540.29 (3104.27–5972.21)	0.001
SHBG (nM)	52.35 (38.88–73.55)	38.20 (23.15–65.30)	0.001	31.90 (18.45–41.50)	20.75 (13.70–30.00)	0.001
Estradiol (pg/mL)	45.50 (37.75–70.00)	67.50 (42.75–96.50)	0.676	45.00 (30.50–68.00)	49.50 (35.00–68.00)	0.339
Progesterone (ng/mL)	0.54 (0.38–0.90)	0.65 (0.45–1.11)	0.017	0.60 (0.44–0.89)	0.67 (0.42–0.91)	0.995
LH (mIU/mL)	4.11 (3.57–6.26)	14.76 (9.40–18.74)	0.562	4.37 (2.48–5.86)	9.48 (5.90–13.12)	0.001
Prolactin (ng/mL)	14.47 (9.91–17.64)	10.70 (7.80–15.62)	0.009	13.14 (10.43–17.11)	10.63 (7.79–15.14)	0.010
FSH (mIU/mL)	7.29 (6.12–9.01)	7.28 (5.79–8.73)	0.026	7.26 (5.94–8.53)	6.60 (5.57–7.67)	0.082
TSH (μIU/mL)	2.09 ± 0.89	2.21 ± 1.18	0.585	2.15 ± 1.25	2.60 ± 4.60	0.536
Total cholesterol (mM)	4.37 (3.95–5.07)	4.41 (4.07–5.20)	0.096	4.54 (4.27–5.01)	4.88 (4.33–5.51)	0.067
HDL (mM)	1.50 (1.30–1.81)	1.36 (1.10–1.57)	0.832	1.15 (0.99–1.36)	1.11 (0.95–1.27)	0.314
LDL (mM)	2.52 (2.25–3.07)	2.63 (2.29–3.08)	0.017	2.87 (2.56–3.25)	3.18 (2.60–3.63)	0.084
Triglycerides (mM)	0.81 (0.60–1.11)	1.02 (0.55–1.64)	0.009	1.14 (0.84–1.84)	1.53 (1.05–2.16)	0.033
FPG (mM)	5.12 (4.89–5.45)	5.15 (4.97–5.34)	0.895	5.31 (4.98–5.67)	5.36 (5.00–5.78)	0.675
FSI (mIU/L)	8.30 (5.98–10.78)	8.30 (5.80–10.75)	0.164	13.10 (9.45–18.5)	16.80 (11.08–21.95)	0.050

BMI, body mass index; BFP, body fat percentage; AMH, anti-Müllerian hormone; Total T, total testosterone; Free T, free testosterone; DHEAS, dehydroepiandrosterone sulfate; SHBG, sex hormone-binding globulin; LH, luteinizing hormone; FSH, follicle-stimulating hormone; TSH, thyroid-stimulating hormone; HDL, high-density lipoprotein cholesterol; LDL, low-density lipoprotein cholesterol; FPG, fasting plasma glucose; FSI, fasting serum insulin. Mean ± standard deviation or median (interquartile range) are shown. The Student’s t test was used for normal distribution data and the Mann–Whitney U test was used for non-normal distribution data.

The PCOS group was divided into hyper-AMH and normal-AMH subgroups. As shown in **Table 2**, the hyper-AMH subgroup had a lower BFP level than the normal-AMH subgroup, but no significant differences were observed between the hyper-AMH subgroup and control group. BFP was greatest in the normal-AMH PCOS subgroup and lowest in the control group.

**Table 2:** Description of the study participants categorized by AMH.

	Control (n = 87)	Hyper-AMH PCOS (n = 58)	Normal-AMH PCOS (n = 98)	*P^a^-*value	*P^b^-*value	*P^c^-*value
Age (year)	31.11 ± 3.27	30.10 ± 2.32	30.45 ± 3.48	0.091	0.451	0.841
BMI (kg/m^2^)	23.21 ± 3.75	24.29 ± 3.94	25.65 ± 3.62	0.276	0.001	0.087
BFP (%)	30.31 ± 5.51	32.27 ± 5.51	35.24 ± 5.44	0.108	0.001	0.004
Waist-hip ratio	0.86 (0.84–0.92)	0.89 (0.85–0.93)	0.92 (0.88–0.96)	0.414	0.001	0.028
Fat mass (kg)	19.17 ± 7.00	20.39 ± 7.32	24.28 ± 7.55	0.986	0.001	0.004
Visceral fat (kg)	9.40 (7.80–11.10)	10.00 (8.88–11.70)	11.25 (9.50–14.00)	0.374	0.001	0.024
AMH (ng/mL)	3.86 ± 2.54	13.77 ± 4.00	5.76 ± 2.09	0.001	0.001	0.001
Total T (ng/mL)	0.46 ± 0.19	0.76 ± 0.24	0.59 ± 0.23	0.001	0.001	0.001
Free T (nM)	0.02 (0.02–0.03)	0.04 (0.03–0.05)	0.03 (0.02–0.04)	0.001	0.003	1.117
DHEAS (nM)	2485.70 (1849.69–3375.52)	4554.60 (3420.58–6248.12)	3983.23 (2768.74–5681.84)	0.001	0.001	0.326
SHBG (nM)	41.60 (27.70–58.40)	28.40 (15.83–39.25)	22.40 (14.70–34.83)	0.001	0.001	1.000
Estradiol (pg/mL)	45.00 (36.00–68.00)	55.00 (42.75–78.75)	52.50 (34.00–78.25)	1.000	0.194	0.804
Progesterone (ng/mL)	0.58 (0.39–0.89)	0.65 (0.45–0.85)	0.66 (0.43–1.05)	1.000	0.710	0.165
LH (mIU/mL)	4.24 (2.92–6.10)	12.24 (9.50–17.20)	9.39 (5.14–14.11)	0.001	0.001	0.002
Prolactin (ng/mL)	13.44 (10.26–17.47)	8.88 (6.89–12.73)	12.22 (8.73–15.74)	0.166	0.001	0.005
FSH (mIU/mL)	7.26 (6.10–8.75)	6.85 (5.98–8.08)	6.70 (4.96–8.13)	0.242	0.310	0.447
TSH (μIU/mL)	2.12 ± 1.07	2.07 ± 1.28	2.71 ± 1.75	1.000	0.599	0.659
Total cholesterol (mM)	4.50 (4.09–5.03)	4.72 (4.09–5.42)	4.71 (4.17–5.47)	0.802	0.240	1.000
HDL (mM)	1.36 (1.12–1.57)	1.27 (1.08–1.51)	1.11 (0.93–1.36)	0.734	0.001	0.006
LDL (mM)	2.73 (2.37–3.10)	2.99 (2.41–3.50)	2.92 (2.44–3.57)	0.706	0.105	1.000
Triglycerides (mM)	0.98 (0.66–1.38)	1.30 (0.86–1.69)	1.48 (0.96–2.14)	0.109	0.001	0.321
FPG (mM)	5.18 (4.93–5.61)	5.26 (5.05–5.58)	5.19 (4.98–5.68)	1.000	1.000	1.000
FSI (mIU/L)	9.70 (6.70–14.60)	9.20 (6.90–14.33)	14.65 (10.50–21.63)	1.000	0.001	0.001

BMI, body mass index; BFP, body fat percentage; AMH, anti-Müllerian hormone; Total T, total testosterone; Free T, free testosterone; DHEAS, dehydroepiandrosterone sulfate; SHBG, sex hormone-binding globulin; LH, luteinizing hormone; FSH, follicle-stimulating hormone; TSH, thyroid-stimulating hormone; HDL, high-density lipoprotein cholesterol; LDL, low-density lipoprotein cholesterol; FPG, fasting plasma glucose; FSI, fasting serum insulin. Mean ± standard deviation or median (interquartile range) are shown. P^a^, comparing the control group and hyper-AMH PCOS group. P^b^, comparing the control group and normal-AMH PCOS group. P^c^, comparing the hyper-AMH group and normal-AMH PCOS group. P-values were adjusted for multiple testing using the Bonferroni method and shown after post-hoc test.

### Serum AMH Showed a Negative Relationship With BFP in PCOS Patients

**Figure 2:**
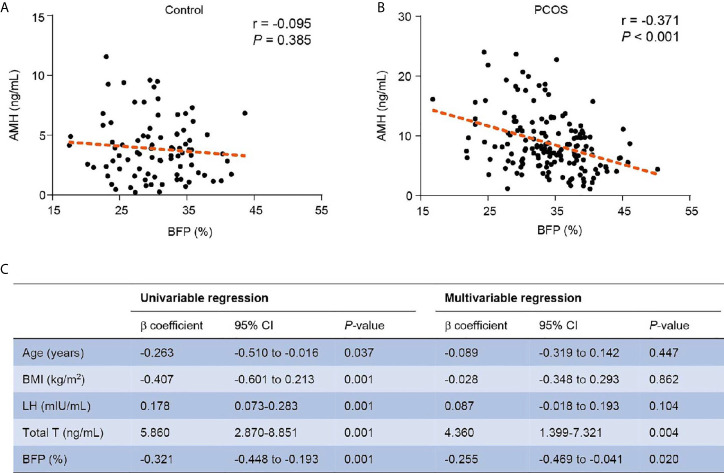
Relationship of BFP with AMH. **(A)** Relationship of BFP with AMH in the control group. **(B)** Relationship of BFP with AMH in the PCOS group. **(C)** Linear regression of BFP with AMH in PCOS patients. PCOS, polycystic ovary syndrome; AMH, anti- Müllerian hormone; BMI, body mass index; LH, luteinizing hormone; Total T, total testosterone; BFP, body fat percentage; CI, confidence interval.

Because AMH was lower in the overweight/obese subgroup of the PCOS and BFP was lower in the hyper-AMH PCOS subgroup, correlation analysis was used to examine the relationship between AMH levels and BFP in PCOS patients. As shown in **Figure 2**, AMH levels were negatively associated with BFP in the PCOS group (r = -0.371, *P* < 0.001). However, BFP was not associated with AMH in the non-PCOS group (r = -0.095, *P* < 0.385). Univariate linear regression and multivariable linear regression models were used to study the effect of BFP on AMH. BFP was associated with AMH in the model (β coefficient -0.255; 95% confidence interval, -0.469 to -0.041; P = 0.020) after adjusting age, BMI, LH, and total testosterone (**Figure 2C**).

### Diagnostic Performance

**Figure 3:**
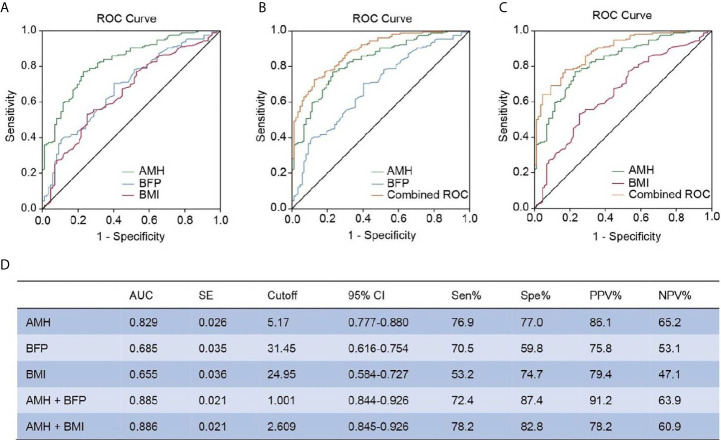
ROC analysis between AMH, BFP, and BMI. **(A)** Diagnostic potential of AMH, BFP, and BMI for PCOS estimated using ROC analysis. **(B)** Combined curve of AMH with BFP. **(C)** Combined curve of AMH with BMI. **(D)** AUC, SE, cutoff value, 95% CI, Sen%, Spe%, PPV%, and NPV% of ROC curves. ROC, receiver operating characteristic; AUC, area under the curve; SE, standard error; CI, confidence interval; Sen%, sensitivity %; Spe%, specificity %; PPV, positive predictive value; NPV, negative predictive value.

Because BFP was markedly elevated in PCOS patients, we used multivariable logistic regression to test the association between BFP and PCOS after adjusting AMH, total testosterone, FSH, LH, fasting serum insulin, and BMI. This analysis revealed that elevated BFP was associated with a high risk of PCOS (P = 0.004; odds ratio, 1.290; 95% confidence interval, 1.084–1.534 (**Table 3**). Finally, ROC curves were used to examine the performance of AMH and BFP. Serum AMH had an AUC of 82.9% at a cutoff value of 5.17 ng/mL, with a sensitivity of 76.9% and a specificity of 77.0%; the positive predictive value (PPV) was 86.1%, and the negative predictive value (NPV) was 65.2%. BFP had an AUC of 68.5% at a cutoff value of 31.45%, with a sensitivity of 70.5% and a specificity of 59.8%; the PPV was 75.8% and the NPV was 53.1%. BMI had an AUC of 65.5% at a cutoff value of 24.95%, with a sensitivity of 53.2%, and a specificity of 74.7%; the PPV was 79.4%, and the NPV was 47.1% (**Figures 3A, D** **)**. The combination of serum AMH and BFP into a multivariate model had an AUC of 88.5%, with a sensitivity of 72.4% and a specificity of 87.4%; the PPV was 91.2% and the NPV was 63.9% (**Figures 3B, D** **)**. The combined curve of serum AMH and BMI had an AUC of 88.6%, with a sensitivity of 78.2% and a specificity of 82.8%; the PPV was 78.2%, and the NPV was 60.9% (**Figures 3C, D** **)**.

**Table 3:** Multivariable logistic regression analysis.

Variable	OR	95CI %	*P*-value
AMH (ng/mL)	1.429	1.208–1.690	0.001
Total testosterone (ng/mL)	8.697	1.219–62.036	0.031
FSH (mIU/mL)	0.827	0.662–1.033	0.094
LH (mIU/mL)	1.306	1.166–1.462	0.001
FSI (mIU/L)	1.063	1.001–1.128	0.045
BMI (kg/m^2^)	0.903	0.696–1.171	0.442
BFP (%)	1.290	1.084–1.534	0.004

AMH, anti-Müllerian hormone; FSH, follicle-stimulating hormone; LH, luteinizing hormone; FSI, fasting serum insulin; BMI, body mass index; BFP, body fat percentage. OR, odds ratio; CI, confidence interval. P-value was calculated by multivariate logistic regression.

## Discussion

To the best of our knowledge, our study is the first to find a negative correlation between BFP and serum AMH levels in women with PCOS, but not in those without PCOS. These observations suggest a link between body fat and AMH metabolism in PCOS patients.

Obesity is present in varying degrees (30%–70%) in women with PCOS (17) and aggravates endocrine and metabolic disorders in individuals with PCOS (18). Pigny et al. found that mean serum AMH levels tended to be lower in obese individuals than in non-obese controls (19). A previous study showed that excess obesity may negatively affect AMH production at the level of the granulosa cells directly (20). Furthermore, some researchers have reported that the oocyte yield is lower in obese women, and that obese women have a higher rate of cycle cancellation (21, 22). Nowadays, obesity is described by BMI worldwide, and several studies have reported an inverse relationship between BMI and AMH, positive association (23), inverse relationship in PCOS (24, 25), or no association (26). Jaswa et al. reported that decreased AMH production by the follicle unit may be responsible for reduced AMH with increasing BMI (6). Thomson et al. reported that weight loss did not change the levels of AMH (27); however, Nilssion-Condori et al. showed that AMH is significantly reduced 1 year after massive weight-loss induced by Roux-en-Y gastric bypass (28). All of these studies explored the relationship and mechanism between AMH and weight. However, none of the previous studies have investigated whether AMH is associated with BFP, and the direct relationship between AMH and BFP remains largely unknown. In addition, no study has focused on the potential mechanism of AMH and fat metabolism. Research has shown a significant interaction between PCOS and BFP (29). The present study found that BFP was higher in PCOS patients compared with control patients despite similar BMI, suggesting that PCOS patients have more fat compared with non-PCOS patients. Research has demonstrated that women with PCOS are more likely to have upper-body fat distribution compared with weight-matched controls (30). In our study, we found that the waist–hip ratio was greater in lean PCOS patients than in the lean control group, suggesting that fat accumulated in the abdomen of lean PCOS patients although they had thin figures. A further novel finding from this study is that the hyper-AMH PCOS patients did not have higher BFP than non-PCOS patients, contrary to the general understanding that obesity is positively associated with PCOS (31). These observations suggest a link between AMH and fat metabolism. However, whether and how obesity and body size might influence ovarian reserve is unclear, and the relationship between AMH, among the indicators of ovarian reserve, and BFP, among the indicators of obesity, remains unknown.

Obesity is associated with PCOS, but its causal role has yet to be determined (31). It is also unclear whether the association between BFP and PCOS primarily relates to PCOS status in particular or to greater visceral obesity or total fat mass. AMH levels have been observed to be higher in obese PCOS versus non-PCOS female adolescents of comparable age and puberty, and thus AMH level may be a useful biomarker for PCOS diagnosis in obese female adolescents (32). Additionally, there is increasing interest in a further putative role for AMH in the pathogenesis of PCOS, acting as an endocrine signal to directly increase gonadotropin-releasing hormone pulse (33, 34). Limitation of our study include the need for larger numbers in both groups, i.e., PCOS and non-PCOS patients. Larger epidemiologic studies are required to confirm our results.

In summary, the present study showed differences in BFP in adult women with and without PCOS. AMH levels were negatively associated with BFP in PCOS patients. Future metabolic studies are needed because the potential mechanism linking BFP and AMH might lead to important insights into ovarian physiology in PCOS patients.

## Data Availability Statement

The raw data supporting the conclusions of this article will be made available by the authors, without undue reservation.

## Ethics Statement

The studies involving human participants were reviewed and approved by Declaration of Helsinki and was approved by the Ethical Review Board at China Medical University. The patients/participants provided their written informed consent to participate in this study.

## Author Contributions

YF conceived and designed the study. EL, JZ, JS, DF, YM, HJ, and DL performed data acquisition and interpretation. YF, JZ, and EL wrote the paper. All authors contributed to the article and approved the submitted version.

## Funding

This work was supported by the National Natural Science Foundation of China (81671423).

## Conflict of Interest

The authors declare that the research was conducted in the absence of any commercial or financial relationships that could be construed as a potential conflict of interest.

## Supplementary Material

Supplementary Table 1 Characteristics of the study participants. BMI, body mass index; BFP, body fat percentage; AMH, anti-Müllerian hormone; Total T, total testosterone; Free T, free testosterone; DHEAS, dehydroepiandrosterone sulfate; SHBG, sex hormone-binding globulin; LH, luteinizing hormone; FSH, follicle-stimulating hormone; TSH, thyroid-stimulating hormone; HDL, high-density lipoprotein cholesterol; LDL, low-density lipoprotein cholesterol; FPG, fasting plasma glucose; FSI, fasting serum insulin. Mean ± standard deviation or median (interquartile range) are shown. The Student’s t test was used for normal distribution data and the Mann–Whitney U test was used for non-normal distribution data.
