# Supplementary figures and images for: Serum Anti-Müllerian Hormone Levels Were Negatively Associated With Body Fat Percentage in PCOS Patients
Source: Front Endocrinol (Lausanne). 2021 Jun 4;12:659717. doi: 10.3389/fendo.2021.659717 (PMC8213015; doi:10.3389/fendo.2021.659717)

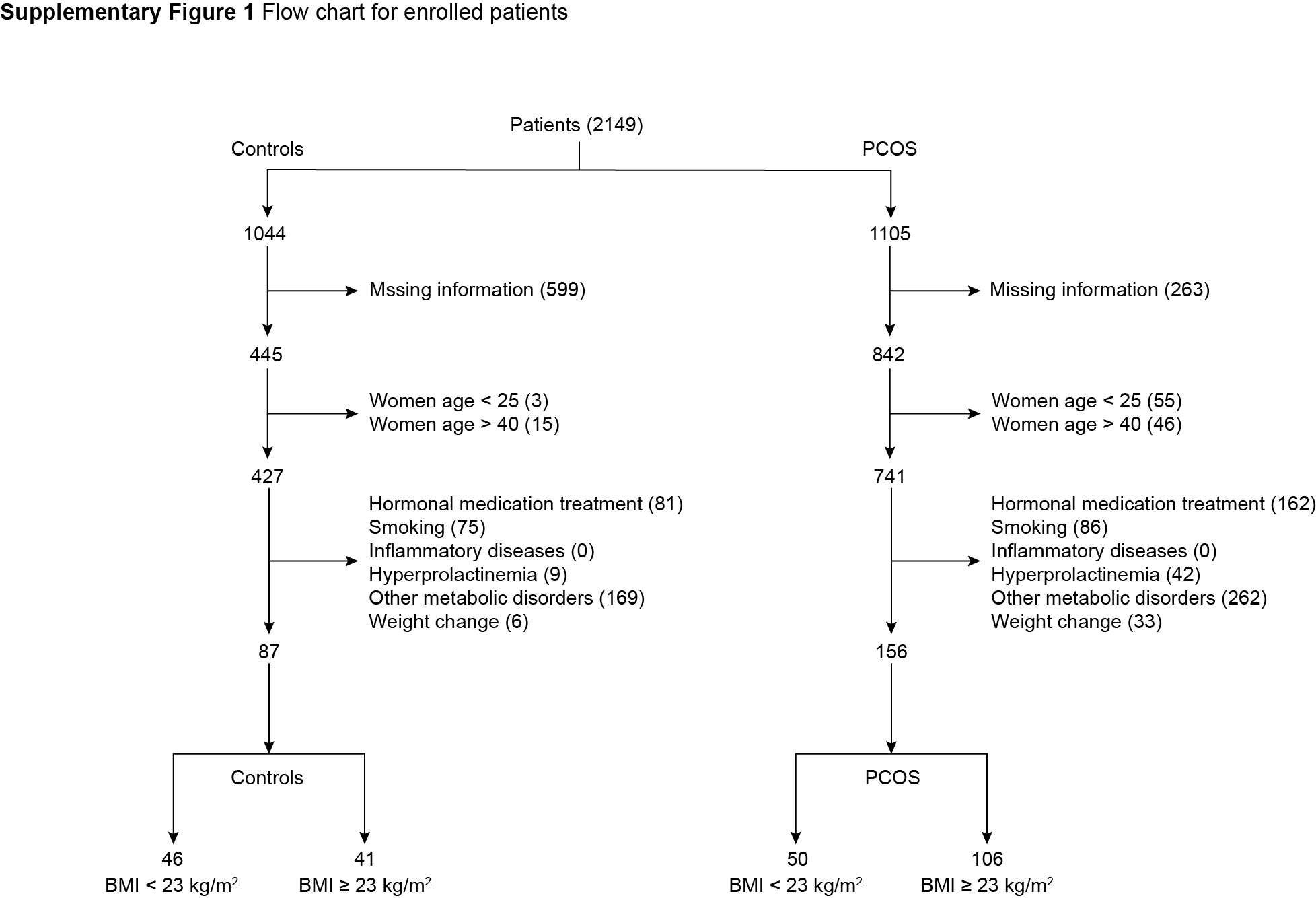

Supplement: Supplementary Table 1 — Characteristics of the study participants. BMI, body mass index; BFP, body fat percentage; AMH, anti-Müllerian hormone; Total T, total testosterone; Free T, free testosterone; DHEAS, dehydroepiandrosterone sulfate; SHBG, sex hormone-binding globulin; LH, luteinizing hormone; FSH, follicle-stimulating hormone; TSH, thyroid-stimulating hormone; HDL, high-density lipoprotein cholesterol; LDL, low-density lipoprotein cholesterol; FPG, fasting plasma glucose; FSI, fasting serum insulin. Mean ± standard deviation or median (interquartile range) are shown. The Student’s t test was used for normal distribution data and the Mann–Whitney U test was used for non-normal distribution data. [file Image_1.jpeg]
