# Supplementary material for: Serum Anti-Müllerian Hormone Levels Were Negatively Associated With Body Fat Percentage in PCOS Patients
Source: Front Endocrinol (Lausanne). 2021 Jun 4;12:659717. doi: 10.3389/fendo.2021.659717 (PMC8213015; doi:10.3389/fendo.2021.659717)
Supplement: Supplementary file 2 [file Table_1.docx]

**Supplementary Table 1** Characteristics of the study participants

|  | **Control (n = 87)** | **PCOS (n = 156)** | ***P*-value** |
| --- | --- | --- | --- |
| Age (year) | 31.11 ± 3.27 | 30.32 ± 3.09 | 0.061 |
| BMI (kg/m^2^) | 23.21 ± 3.75 | 25.14 ± 3.79 | 0.001 |
| BFP (%) | 30.31 ± 5.51 | 34.13 ± 5.63 | 0.001 |
| Waist-hip ratio | 0.86 (0.84–0.92) | 0.91 (0.87–0.95) | 0.001 |
| Fat mass (kg) | 19.17 ± 7.00 | 22.83 ± 7.67 | 0.001 |
| Visceral fat (kg) | 9.40 (7.80–11.10) | 11.00 (9.00–13.10) | 0.001 |
| AMH (ng/mL) | 3.86 ± 2.54 | 8.74 ± 4.87 | 0.001 |
| Total T (ng/mL) | 0.46 ± 0.19 | 0.65 ± 0.25 | 0.001 |
| Free T (nM) | 0.02 (0.02–0.03) | 0.03 (0.02–0.05) | 0.001 |
| DHEAS (nM) | 2485.70  (1849.69–3375.52) | 4172.31  (3006.54–5847.68) | 0.001 |
| SHBG (nM) | 41.60 (27.70–58.40) | 23.75 (15.33–37.38) | 0.001 |
| Estradiol (pg/mL) | 45.00 (36.00–68.00) | 53.00 (38–78) | 0.091 |
| Progesterone (ng/mL) | 0.58 (0.39–0.89) | 0.66 (0.43–1.00) | 0.275 |
| LH (mIU/mL) | 4.24 (2.92–6.10) | 10.74 (6.37–15.62) | 0.001 |
| Prolactin (ng/mL) | 13.44 (10.26–17.47) | 10.65 (7.8–15.17) | 0.001 |
| FSH (mIU/mL) | 7.26 (6.10–8.75) | 6.74 (5.61–8.13) | 0.037 |
| TSH (μIU/mL ) | 2.12 ± 1.07 | 2.47 ± 3.85 | 0.278 |
| Total cholesterol (mM) | 4.50 (4.09–5.03) | 4.71 (4.14–5.43) | 0.048 |
| HDL (mM) | 1.36 (1.12–1.57) | 1.18 (0.97–1.38) | 0.001 |
| LDL (mM) | 2.73 (2.37–3.10) | 2.93 (2.43–3.51) | 0.033 |
| Triglycerides (mM) | 0.98 (0.66–1.38) | 1.37 (0.94–1.96) | 0.001 |
| FPG (mM) | 5.18 (4.93–5.61) | 5.22 (4.99–5.67) | 0.336 |
| FSI (mIU/L) | 9.70 (6.70–14.60) | 12.05 (9.03–19.08) | 0.002 |

**Abbreviations:** BMI, body mass index; BFP, body fat percentage; AMH, anti-Müllerian hormone; Total T, total testosterone; Free T, free testosterone; DHEAS, dehydroepiandrosterone sulfate; SHBG, sex hormone-binding globulin; LH, luteinizing hormone; FSH, follicle-stimulating hormone; TSH, thyroid-stimulating hormone; HDL, high-density lipoprotein cholesterol; LDL, low-density lipoprotein cholesterol; FPG, fasting plasma glucose; FSI, fasting serum insulin. Mean ± standard deviation or median (interquartile range) are shown. The Student’s t test was used for normal distribution data and the Mann–Whitney U test was used for non-normal distribution data.
